# Cell wall remodeling promotes callus formation in poplar

**DOI:** 10.1186/s43897-024-00093-4

**Published:** 2024-05-08

**Authors:** Geng Zhang, Peipei Liu, Guifang Zhang, Xiaomin Yao, Xinwei Wang, Yueqian Zhang, Jinxing Lin, Yaning Cui, Xiaojuan Li

**Affiliations:** 1 State Key Laboratory of Efficient Production of Forest Resources, College of Biological Sciences and Technology, Beijing Forestry University, Beijing, 10083 China; 2 Key Laboratory of Genetics and Breeding in Forest Trees and Ornamental Plants, Ministry of Education, Beijing Forestry University, Beijing, 100083 China; 3 College of Agriculture and Forestry, Hebei North University, Zhangjiakou, 075000 China

In plant tissue culture, callus, a group of pluripotent cells, can be induced from detached explants by phytohormones on callus-inducing medium (CIM), and then callus can differentiate into root or shoot tissues, respectively (Xu et al. 2019; Zhai et al. 2024). Therefore, callus formation is thought to reflect a change in cell fate whereby differentiated somatic cells reacquire pluripotency (Xu et al. 2019). Various studies have uncovered the molecular mechanisms underlying changes in cell fate during callus formation (Zhai and Xu 2021). In addition to changes in gene expression, cell wall changes are also involved in regulating plant regeneration (Xu et al. 2019). For instance, cell wall damage activates four transcription factor genes from the DOF (DNA binding with one finger) family that promote tissue regeneration (Zhang et al. 2022). Recent research has also shown that the expression of cell-wall-loosening enzymes in the shell of cells surrounding the progenitor can activate cell polarity in progenitors to promote shoot regeneration from an undifferentiated callus (Varapparambath et al. 2022). However, the mechanisms underlying how the cell wall participates in callus formation remain unclear, especially in woody plants.

To investigate the molecular basis of differentiated cells reacquiring pluripotency during callus formation, we performed the whole transcriptome analysis of 84 K poplar (*Populus alba x P. tremula* var. *glandulosa*) during the three stages of callus formation (Fig. 1A). We identified 28,984 messenger RNAs (mRNAs), 1,719 long non-coding RNAs (lncRNAs), 1171 novel circular RNAs (circRNAs), and 540 microRNAs (miRNAs) (Supplementary Table S1). To further explore gene expression patterns during callus formation, we identified differentially expressed (DE) mRNA and ncRNA at the three stages using the criteria |Log_2_(foldchange) |> 1 and *p* < 0.05 through pairwise comparisons (Supplementary Table S2, Supplementary Fig. 1A). The expression profiles of DE RNAs at the three time points were plotted using a clustering heatmap (Fig. 1B, Supplementary Fig. 1B). The key genes involved in cell fate modulation, such as *WOX5, WOX11, PLT1*, and *LBD16*, were upregulated in stage S2 compared to S1 (Supplementary Table S3). The results were consistent with previous reports (Xu et al. 2019), indicating their crucial role in altering cell fate during callus induction. During the phase from S2 to S3, the expressions of reprogramming-related genes did not significantly change, and the genes that maintained the identity of callus cells and promoted cell division, such as *LBD16*, *LBD29* and *WOX11* (Liu et al. 2018)*,* were highly expressed. As a result, we categorized pluripotency acquisition into two distinct phases: cell fate transition (S1 vs. S2) and proliferation (S2 vs. S3).

**Fig. 1:**
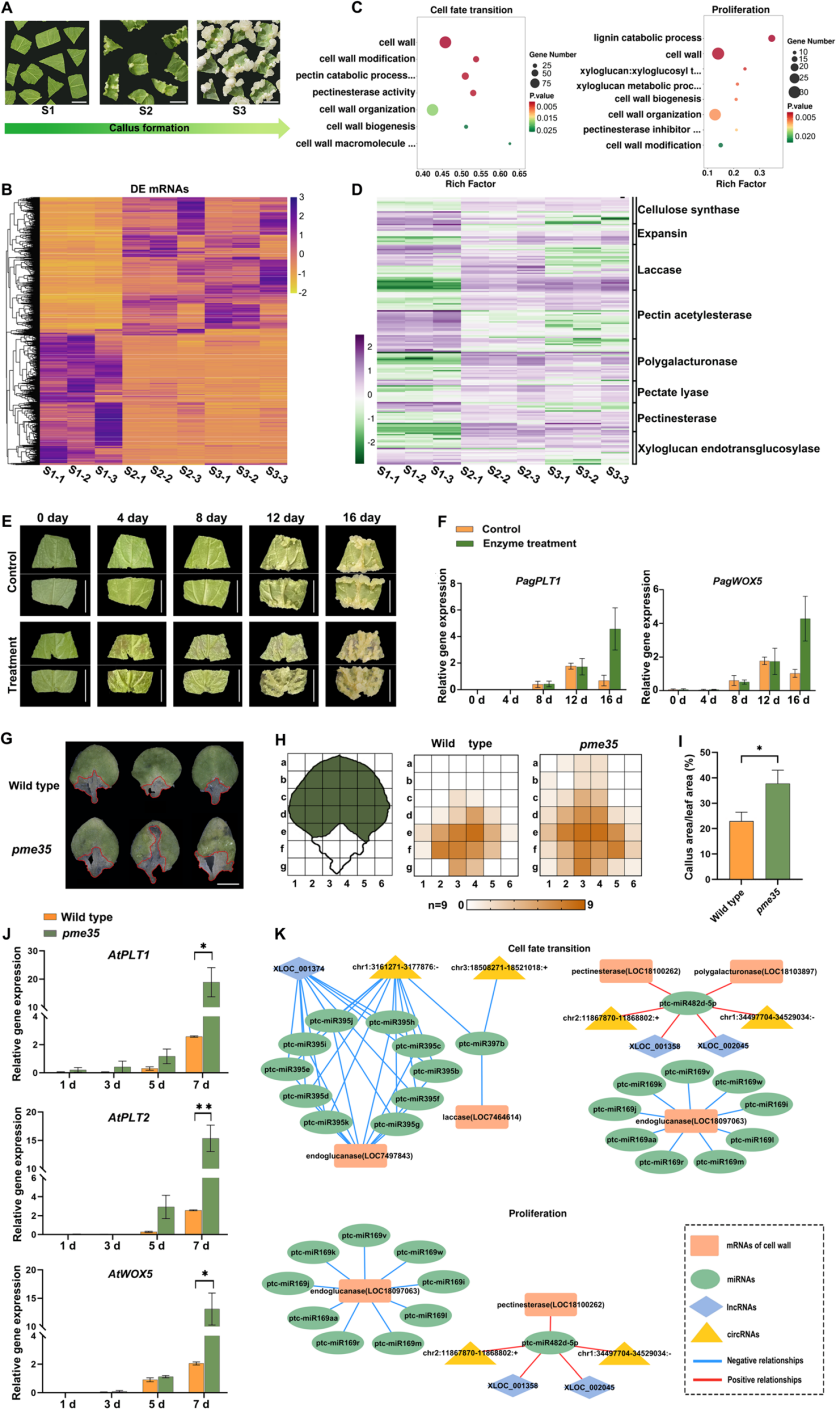
**A** Plant material used for whole transcriptome sequencing. Scale bars, 1 cm. **B** Heatmap showing the transcript levels of differentially expressed genes. Transcript levels (average FPKM) were Log_10_-normalized. Purple, upregulation; orange, downregulation. **C** Cell wall-related GO term enrichment of differentially expressed mRNAs in cell fate transition and proliferation. The size of the circle indicates the number of genes and the color indicates the p-value. **D** Heatmap showing the transcript levels of genes related to the cell wall. Transcript levels (average FPKM) were Log_10_-normalized. Purple, upregulation; green, downregulation. **E** Callus-forming phenotypes of poplar leaves on 0, 4, 8, 12 and 16 days cultured on CIM. Scale bars, 1 cm. **F** qRT-PCR analysis showing *PagPLT1* and *PagWOX5* transcript levels during tissue culture of poplar explants. **G** The callus-forming phenotype of wild-type and *pme35* plants cultured on CIM for 10 days, with callus marked in red. Scale bars, 2 mm. **H** Heatmap showed the number of explants forming callus on each part of wild type and *pme35* when cultured on CIM for 10 days (*n* = 9). **I** Relative ratio of callus area to the total leaf area at 10 days. Data are shown as mean ± SEM (*n* = 9) (*P < 0.05, Student’s t-test). **J** qRT–PCR analysis of transcript levels for *AtPLT1*, *AtPLT2*, and *AtWOX5* during tissue culture of wild-type and *pme35* leaf explants on CIM. Data are means ± SEM from three biological replicates. Each biological replicate contained three technical replicates. Data are shown as mean ± SEM (*P < 0.05, **P < 0.01; Student’s t-test). **K** Cell wall-related ceRNA network in cell fate transition and proliferation phases

To better understand the mechanisms involved in callus formation, we performed functional enrichment analyses of DE mRNAs utilizing Gene Ontology (GO) databases. The enriched GO terms were associated with biosynthetic processes, catabolic processes, and defense responses (Supplementary Fig. 1C, Supplementary Table. S4). Among the various biological processes implicated by enriched GO categories, significant enrichment of cell wall biogenesis, modification, and macromolecule catabolic processes was detected during callus formation (Fig. 1C, Supplementary Table. S4). Therefore, we focused on the functions and dynamic changes of the GO categories associated with the cell wall. The expression of genes associated with cell wall degradation and loosening was predominantly upregulated during callus formation, including those coding for pectate lyase, pectin esterase/acetyl esterase, polygalacturonase, xyloglucan endotransglucosylase, and expansin (Fig. 1D, Supplementary Table S5). mRNAs related to cell wall biosynthesis, such as those encoding for cellulose synthase-like proteins, also exhibited dynamic expression patterns during callus formation (Fig. 1D, Supplementary Table S5). Based on these results, we hypothesized that cell wall degradation and biosynthesis were required for cell fate transition.

In order to further elucidate the role of cell wall integrity in callus formation, we treated 84 K poplar leaves with pectinase and cellulase. A large amount of calli was detected to form in most of the vascular bundles of explants treated with wall-degrading enzymes. However, calli were only observed at the incision and the midvein of untreated explants after culturing on CIM for 16 days (Fig. 1E). Moreover, the expression of *PagPLT1* and *PagWOX5,* which serve as key regulators in callus formation (Zhai and Xu 2021), increased from day 8 to day 12 both in control and in enzymatically treated leaf explants (Fig. 1F). However, the tendency for changes was different from day 12 to day 16. In the control explants, the expression levels of *PagPLT1* and *WOX5* on day 16 were lower than those on day 12. Whereas, in explants treated with enzymes, the expression levels of *PagPLT1* and *WOX5* on day 16 were higher than those on day 12, an increase of 2.7-fold and 2.5-fold, respectively (Fig. 1F), suggesting that *PagWOX5* and *PagPLT1* were consistently upregulated in enzymatically treated explants. Furthermore, we transferred the calli derived from enzyme-treated and control poplar explants to shoot-inducing medium (SIM). After 60 days, regenerated shoots were observed from both treated and control calli (Supplementary Fig. 1D), demonstrating the pluripotency of these calli. These results suggest that damage to the cell wall positively regulates callus formation.

Then we detected the expression of genes involved in cellulose, hemicellulose, and pectin and analyzed their transcript levels (Supplementary Table S6) according to previous studies (Du et al., 2020; Tang et al. 2022). Notably, the expression of *PagPME35* continuously decreased during callus formation (Supplementary Table S6). In Arabidopsis, the homologous gene *AtPME35* encodes a pectin methylesterase, contributing to the strengthening of cell walls (Hongo et al. 2012). Furthermore, more calli were detected on explants from Arabidopsis *pme35* mutants compared to the wild-type, and the expression of *PLT1, PLT2,* and *WOX5* was upregulated more rapidly in *pme35* (Fig. 1J). Importantly, the calli derived from *pme35* had the ability to regenerate shoots, similar to the calli from wild type explants (Supplementary Fig. 1E). These results suggest that cell wall properties, in turn, regulate developmental programs.

To better understand the complex regulatory network of mRNAs and ncRNAs related to the cell wall, we constructed competing endogenous RNA (ceRNA) networks based on ceRNA theory. The ceRNA theory posits that mRNAs, lncRNAs, and circRNAs can interact with miRNAs through competitive binding to a common miRNA-binding site (Teng et al. 2023). Using target prediction and expression correction analysis, we identified 339 DE miRNA-DE mRNA, 504 DE miRNA-DE circRNA, and 173 DE miRNA-DE lncRNA interactions (Supplementary Table S7). Subsequently, ceRNA networks were constructed for two distinct phases, utilizing DE mRNAs specifically related to the cell wall (Fig. 1K). During the cell fate transition, some differentially expressed mRNAs (LOC7497843, LOC18103897 and LOC7464614) encoding enzymes, such as endoglucanase, polygalacturonase and laccase, were predicted to be regulated by lncRNAs and circRNAs through miRNAs. For instance, the mRNA (LOC7497843) encoding endoglucanase was upregulated at cell fate transition, and it was predicted to be negatively regulated by lncRNA (XLOC_001374) and circRNA (chr1:3,161,271–3,177,876:-) through the ptc-miR395 family. Since endoglucanases plays a role in cleaving internal β-glycosidic bonds in the cellulose chain (Yennamalli et al. 2013), this result suggests that the interaction of these ncRNAs with the mRNA might be involved in cellulose metabolism. In both networks, the mRNA (LOC18100262) encoding pectinesterase, which participated in the conversion of protopectin to soluble pectin and pectate (Markovic and Jornvall, 1986), was predicted to be negatively regulated by two lncRNAs (XLOC_001358 and XLOC_002045) and two circRNAs (chr2:11867870–11868802: + and chr1:34497704–34529034:-) through the ptc-miR482d-5p, forming a complex network associated with pectin degradation. Additionally, an endoglucanase mRNA (LOC18097063) was predicted to be negatively regulated by the miR169 family in both phases, suggesting that miR169s might affect cell wall metabolism during callus formation. Taken together, these results indicate that regulation of cell wall-related mRNA with tight temporal coordination of ncRNAs would be critical for highly programmed callus formation.

In conclusion, our study provides novel insights into the molecular mechanisms underlying callus formation in poplar, with important implications for the development of improved plant regeneration technology. We profiled mRNA, miRNA, lncRNA, and circRNA expression during callus formation in poplar and established that cell wall-related genes displayed dynamic transcriptional changes. The changes in genes related to cell wall relaxation and remodeling suggest a potential mechanism by which mature cells can be converted into pluripotent cells capable of dividing and forming callus. Moreover, modifying cell walls using enzymatic digestion of cellulose and pectin confirmed the regulatory role of the cell wall on callus formation. The callus-forming phenotypes of *pme35* further highlighted the importance of investigating the role of cell wall modification, indicating that cell wall remodeling during callus formation is not only due to changes in cell mechanical properties but also actively influences callus formation. Additionally, we constructed a cell wall-related lncRNA/circRNA‒miRNA–mRNA regulatory network, which provides a valuable foundation for future studies aimed at elucidating the molecular mechanisms underlying callus formation. Our findings reveal the crucial role of cell wall remodeling in promoting callus formation, laying the groundwork for innovative strategies in callus-based organ regeneration. This has significant implications for the development of plant regeneration technology, particularly for rare or economically valuable tree species.

### Supplementary Information

**Additional file 1:** Materials and Methods.

**Additional file 2:** Supplementary Figure S1.

**Additional file 3: Supplementary Table S1.** All RNAs from S1, S2 and S3 of 84 K poplar. **Supplementary Table S2.** All differential expression RNAs from S1, S2 and S3 of 84 K poplar. **Supplementary Table S3.** Transcription profiles of the genes related to callus formation. **Supplementary Table S4.** GO term enrichment of differentially expressed mRNAs. **Supplementary Table S5.** Differential expression genes associated with cell wall. **Supplementary Table S6**. Transcription profiles of the poplar genes orthologous to Arabidopsis genes involved in cell wall. **Supplementary Table S7.** Pairwise expression correlations of all miRNAs and target RNAs.

**Additional file 4:** Primer sequences used for qRT-PCR.

## Data Availability

The original data used to support the findings can be obtained by contacting the corresponding author. All data supporting this research result can be obtained in the paper and within its Supplementary Materials published online.

## Abbreviations

ceRNA: competing endogenous RNA
circRNA: circular RNA
DE: differentially expressed
lncRNA: long non-coding RNAs
ncRNA: non-coding RNAs
PLT: PLETHORA
WOX: WUSCHEL-RELATED HOMEOBOX
