# Supplementary material for: Cell wall remodeling promotes callus formation in poplar
Source: Mol Hortic. 2024 May 8;4:16. doi: 10.1186/s43897-024-00093-4 (PMC11059702; doi:10.1186/s43897-024-00093-4)
Supplement: Supplementary file 1 — Additional file 1: Materials and Methods. [file 43897_2024_93_MOESM1_ESM.docx]

**Materials and Methods**

**Plant materials and tissue culture**

The 84K poplar (*Populus alba x P. tremula* var*. glandulosa*) was used for callus induction experiment. The 84K poplar were cultivated in half-strength MS medium (1/2 MS medium, 20 g L^−1^ sucrose, 7.8 g L^−1^ agar, pH 5.8) under sterile conditions (16-h-light, 22 ℃) for 30 days. The leaves (third to fifth leaf from the top) were collected. Some of the leaves were stored in -80℃ and the others were cultured on CIM (MS basal medium supplemented with 1 mg L^−1^ 2,4-D, 0.1mg L^−1^ kinetin, 20 g L^−1^ sucrose, and 7.8 g L^−1^ agar, pH 5.8). After incubation for 2 weeks in dark, callus began to appear on the edge of leaf explants. Then the portion of the leaves where callus began to form were collected and stored in -80℃. The remained explants and callus were further cultured for a week. The compact and vigorous callus particles were collected and stored in -80℃.

Arabidopsis Columbia-0 plants were taken as the wild type. The T-DNA insertion mutants *pme35* (SALK_019255C) were obtained from the AraShare (www.arashare.cn) and verified by PCR analyses. For tissue culture, seedlings were grown on 1/2 MS (1/2 MS medium, 20 g L^−1^ sucrose, 7.8 g L^−1^ agar, pH 5.8) at 22°C under a 16-h-light/8-h-dark photoperiod. The first pair of rosette leaves were cut from 12-day-old seedlings and cultured on CIM (MS medium, 20 g L^−1^ sucrose, 0.5 mg L^−1^ 2,4-D, 0.05 mg L^−1^ kinetin, and 7.8 g L^−1^ agar, pH 5.8) to induce callus formation. The callus cultivated on SIM (MS with 10 g L^−1^ zeatin, 5 g L^−1^ d-biotin, 1 g L^−1^ IBA). After incubation on SIM for 1 months, the regenerated shoots on callus were observed.

**RNA high-throughput sequencing and bioinformatics**

RNA-sequencing was performed in Genesky (Shanghai, China). Total RNA was extracted using an RNAprep Pure Plant Kit (TianGen, China). The miRNA of the samples for small RNA-Seq was extracted with an RN40-EASYspin plant microRNA kit (Aidlab, China). The strand-specific libraries and small RNA sequencing libraries were performed on an Illumina HiSeq 2500 platform (Illumina, USA). Sequence quality was evaluated by the FastQC software v0.11.4. By using the HISAT2, clean reads from cDNA libraries were mapped to the *Populus trichocarpa* v3.0 reference genome (https://phytozome.jgi.doe.gov/pz/portal.html). FPKM values (fragments per kilobase of transcript per million fragments mapped) for the mRNAs and ncRNAs were obtained for each sample. The differentially expressed RNAs were analyzed using the R package maSigPro with |log_2_FoldChange|>1 and *P* value < 0.05. The heatmap was performed using the OmicStudio tools at https://www.omicstudio.cn/tool.

GO enrichment analysis of the differentially expressed genes were performed by R Clusterprofiler (<http://bioconductor.org/packages/release/bioc/html/clusterProfiler.html>).

**qRT-PCR**

Total RNA was extracted using the EASYspin plus plant RNA kit (Aidlab, Beijing). Reverse transcription was carried out with the TransScript ⅡOne-step gDNA Removal and cDNA Synthesis SuperMix (Trans, Beijing). The quantitative real-time PCR was performed using the TB Green Premix Ex Taq II (Takara, Dalian) according to the standard protocol on a Bioer 96plus (Bioer, Hanzhou) in a 20 μL reaction volume. The amplification program was 94 °C for 30s, and 45 cycles at 94°C for 5s, and 60°C for 30s. PCRs were performed in triplicate for each sample. The gene-specific primers in this study are listed in Additional file 3.

**Enzymatic digestion of cell wall**

Leaves from 30-day-old 84K poplar were cut and soaked in the enzyme solution (D-Mannitol, 72.86 g L^−1^; MES·H_2_O, 4.26 g L^−1^; KCl, 1.5 g L^−1^; CaCl_2_·2H_2_O, 1.5 g L^−1^; BSA, 1 g L^−1^; pectinase,10 g L^−1^; cellulase, 10 g L^−1^ ) for 1 hour. For control experiments without enzymatic digestion, identical procedures were used except that no pectinase and cellulase was added. Then explants with and without enzyme treatment were washed with sterile water for 1 min, and then cultured on CIM (MS basal medium that was supplemented with 1 mg L^−1^ 2,4-D, 0.1mg L^−1^ kinetin, 20 g L^−1^ sucrose, and 7.8 g L^−1^ agar, pH 5.8) in the dark to induce callus. The callus cultivated on SIM (MS with 0.02mg L^−1^ TDZ) under a 16-h light and 8-h dark photoperiod at 22◦C. After incubation on SIM for 2 months, the regenerated shoots on callus were observed.

**Visualization of callus formation patterns**

Statistical analysis of callus formation patterns was performed as described in the previous report (Shin *et al*., 2022). The images of explants were divided into 6× 7 sections. 9 explants with similar orientations were analyzed. The number of explants showing callus formation on each section were shown as a heatmap using GraphPad Prism.

**Construction of the cell wall-related ceRNA network**

Target RNAs of miRNAs were determined by miRanda (http://www.microrna.org/microrna/home.do) and RNAhybrid (<http://bibiserv.techfak.uni-bielefeld.de/rnahybrid/>). The miRNA- mRNA, miRNA-lncRNA, and miRNA-circRNA pairs were predicted. The ceRNA networks were visualized using Cytoscape software (version 3.7.1).

**References**

Shin SY, Choi Y, Kim S, Park S, Park J, Moon K, Kim H, Jeon JH, Cho HS, Lee H. Submergence promotes auxin-induced callus formation through ethylene-mediated post-transcriptional control of auxin receptors. Mol Plant. 2022; 15(12), 1947-1961. https://doi.org/10.1016/j.molp.2022.11.001
