# Supplementary material for: Cell wall remodeling promotes callus formation in poplar
Source: Mol Hortic. 2024 May 8;4:16. doi: 10.1186/s43897-024-00093-4 (PMC11059702; doi:10.1186/s43897-024-00093-4)
Supplement: Supplementary file 2 — Additional file 2: Supplementary Figure S1. [file 43897_2024_93_MOESM2_ESM.docx]

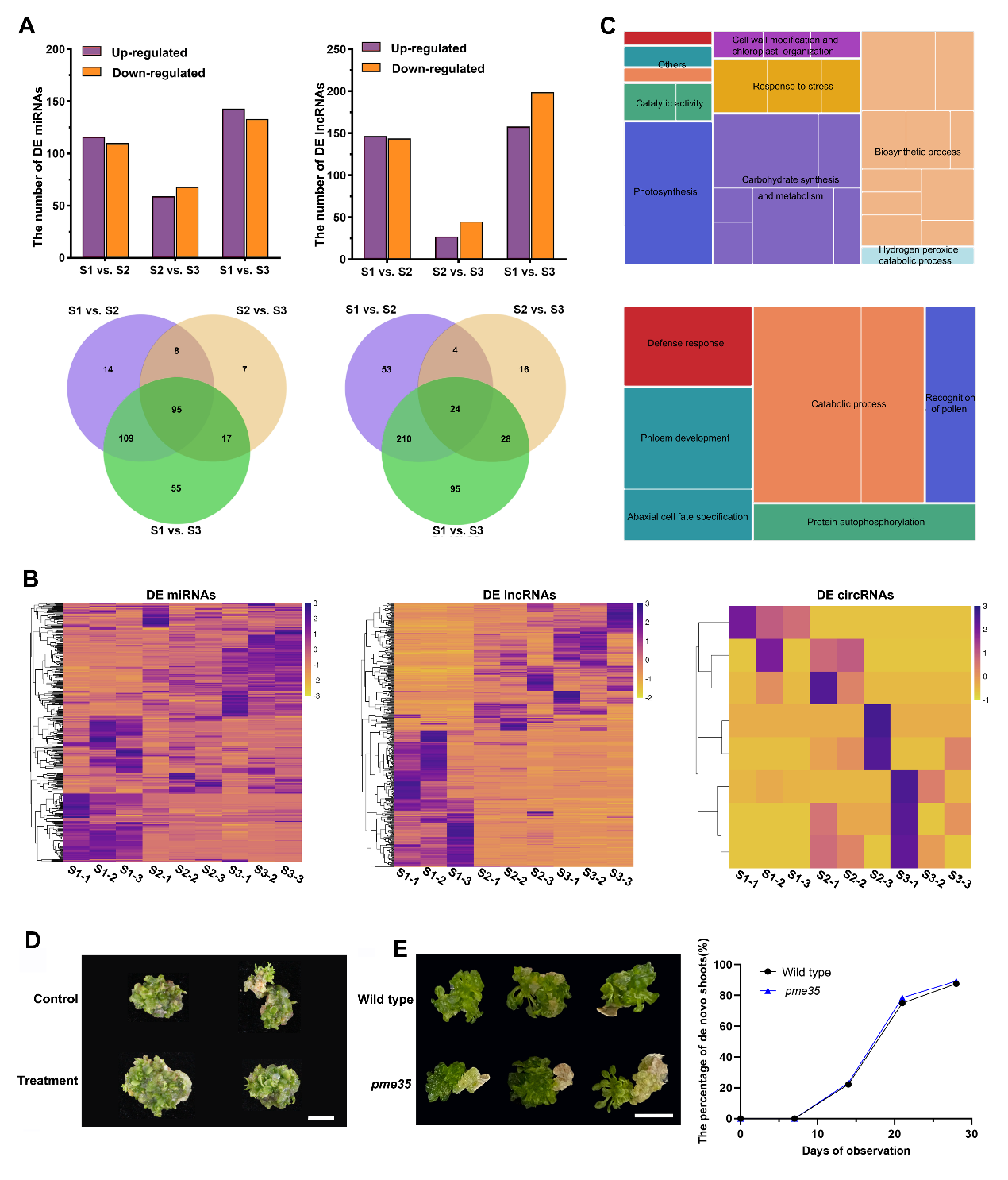


**Fig. S1.** (A) Number of differentially expressed ncRNAs across samples. Venn diagrams showing the extent of overlap between differentially expressed miRNAs and lncRNAs in the three comparison groups. (B) Transcriptome profiles of differentially expressed ncRNAs. Transcript levels (average FPKM) were Log_10_-normalized. Purple, upregulation; orange, downregulation. (C) GO term enrichment of differentially expressed mRNAs in the S1 vs. S2 and S2 vs. S3 comparisons. Only selected GO terms in the category of biological processes are shown. The aggregate size indicates the significance level of the GO term (*p*-adjust <0.05). (D) Shoot-regeneration phenotypes of poplar callus cultured on SIM for 60 d. Scale bars 1 cm. (E) Analysis of shoot-regeneration phenotypes of Arabidopsis callus cultured on SIM for 30 d. Scale bars 1 cm.
