# Supplementary material for: Cell wall remodeling promotes callus formation in poplar
Source: Mol Hortic. 2024 May 8;4:16. doi: 10.1186/s43897-024-00093-4 (PMC11059702; doi:10.1186/s43897-024-00093-4)
Supplement: Supplementary file 4 — Additional file 4: Primer sequences used for qRT-PCR. [file 43897_2024_93_MOESM4_ESM.docx]

**Primer sequences used for qRT-PCR.**

| **Gene Name** | **Forward primers** | **Reverse primers** |
| --- | --- | --- |
| **For *Arabidopsis thaliana*** | | |
| *actin* | AAGCTCTCCTTTGTTGCTGTT | GACTTCTGGGCATCTGAATCT |
| *AtWOX5* | CTGTTTCGAGCCGGTCTTAG | TCACCTTCTCTTCCTCTTGACA |
| *AtPLT1* | TCGAGGAGTTACAAGGCA TCAC | TGCTGCTTCTTCCTCAGTGC |
| *AtPLT2* | ACTTGGGAAC ATTCAGCACGG | AGTGTGTTGCTCTCCAGGATG |
| **For *Populus alba x P. tremula* var*. glandulosa*** | | |
| *18S* | CGAAGACGATCAGATACCGTCCTA | TTTCTCATAAGGTGCTGGCGGAGT |
| *PagWOX5*  *PagPLT1* | AGACAGAAGAGGCGCAGAGT  AGCTAGCTAGGGCCTTCTTTG | CTCAATCACTTGTTCTCGTT  AGTCAGTTCATCTTCTCCGGTT |
